# Heat Losses Caused by the Temporary Influence of Wind in Timber Frame Walls Insulated with Fibrous Materials

**DOI:** 10.3390/ma13235514

**Published:** 2020-12-03

**Authors:** Piotr Kosiński, Przemysław Brzyski, Zbigniew Suchorab, Grzegorz Łagód

**Affiliations:** 1 Faculty of Geoengineering, University of Warmia and Mazury in Olsztyn, Jana Heweliusza 10, 10-724 Olsztyn, Poland; 2 Faculty of Civil Engineering and Architecture, Lublin University of Technology, Nadbystrzycka 40, 20-618 Lublin, Poland; p.brzyski@pollub.pl; 3 Faculty of Environmental Engineering, Lublin University of Technology, Nadbystrzycka 40B, 20-618 Lublin, Poland; z.suchorab@pollub.pl; 4 Faculty of Civil Engineering, Czech Technical University in Prague, Thákurova 7, 166 29 Prague, Czech Republic

**Keywords:** thermal insulation, heat losses, thermal conductivity, air filtration, wind pressure, air permeability, fibrous materials, porous materials, water condensation

## Abstract

The paper presents the results of research concerning three fiber materials—mineral wool, hemp fiber and wood wool—as loose-fill thermal insulation materials. The analysis used the material parameters determined in previous works conducted by the authors, such as thermal conductivity and air permeability in relation to bulk density. These materials exhibit open porosity; thus, convection is an essential phenomenon in the heat transfer process. The paper aimed at conducting thermal simulations of various frame wall variants which were filled with the above-mentioned insulation materials. The simulations were performed with the Control Volume Method using the Delphin 5.8 software. The studies accounted for the effect of wind pressure and the time of its influence on a wall insulated by means of fiber material with a thickness of 150 as well as 250 mm. The simulation enabled us to obtain such data as maximal R-value reduction and time to return to equilibrium after filtration for the analyzed materials. The study proved that heat transfer in these insulations strongly depends on the bulk density, thickness of the insulation and wind pressure. The decrease in R is reduced as the density increases. This results from the decreased air permeability characterizing the material. Wind washing causes lower R reduction than air filtration in all models. The greater the thickness, the longer it takes for the models to return to the equilibrium state following air filtration (and wind washing). This period is comparable for air filtration and wind washing. Hemp fibers were characterized with the strongest susceptibility to air filtration; in the case of wood wool, it was also high, but lower than for hemp fibers, while mineral wool was characterized with the lowest.

## 1. Introduction

Lightweight external walls made in timber frame construction should be designed as wind-resistant. Thermal insulation should tightly fill the space between the elements of the construction and the outside should be covered with the wind barrier. Otherwise, or in case the wind barrier is damaged, the cold air blown by the wind inside the external wall will contribute to the temperature decrease inside the wall as well as on its surface and—in consequence—to the heat losses inside the building and even to the surface condensation as well as the possibility of mold growth on the wall surface. A wind barrier prevents outdoor air infiltration through natural or forced convection in the thermal insulation. However, this air barrier should be permeable enough for water vapor to pass through in order to avoid condensation inside the wall construction, which—in turn—would lead to additional heat losses and corrosion processes that may cause microbiological degradation of timber elements as a consequence. Timber frame constructions are insulated with, i.e., hemp–lime composite, which covers the structure and reduces the thermal bridges [1,2]. However, fiber materials, such as mineral wool, wood wool and hemp fiber, are most often used for insulation purposes, either as loose-fill thermal insulation materials or in the form of mats/slabs. The thermal insulation fiber materials are characterized by varying air permeability, depending on their density [3]. Air infiltration, resulting from the impact of wind blowing through the walls, depends on the surrounding conditions, mainly on the temperature differences and on the atmospheric pressure at both sides of the wall [4,5]. Air infiltration results in excessive heat flow through the walls as well as in changes in the thermal properties of the materials from which the wall is made [6], e.g., their increased thermal conductivity [7]. A review of studies [4] shows that infiltration is responsible for 25–50% of heat loss in buildings. The exact value of losses strongly depends on the thermal insulation level of a building (including the quality and thickness of the insulating material) and the tightness of the building envelope. The heat loss resulting from infiltration is proportional to the amount of air flowing through the wall gaps and is connected with the pressure and the wind velocity [8]. The infiltration also causes increased moisture movement through the wall, which may contribute to water condensation [9,10]. The water present in building structures due to the phenomenon of condensation is an energy carrier and a key factor that decreases the thermal properties of porous materials [11,12,13] but also leads to the mechanical [14,15] and microbiological deterioration of the materials [16,17,18,19].

Heat transfer through the building envelopes is calculated on the basis of the thickness of the envelope layers and the thermal conductivity of the materials comprised by the envelope. Thermal conductivity is one of the mechanisms for transferring the heat flow through the wall. Other mechanisms for heat transfer may be, in some cases, crucial as well, such as forced air infiltration resulting from the wind impact on the wall (wind washing). The heat flow caused by the wind is not taken into account in the standard thermal calculations when designing the walls in residential buildings [20]. Deseyve and Bednar [21] showed that the airflow caused by wind is the main cause of the increasing heat flow in lightweight roof structures. The issue of the airtightness of the building walls was discussed in many studies [22,23,24,25]. The correlation between the energy demand for heat purposes and the building airtightness was investigated. It was concluded that the airtightness of the building is responsible for the velocity of air infiltration, which, in turn, increases the heat loss.

In their research and measurements, Timusk et al. [26] and Uvsløkk [27] demonstrated that there is a clear impact of the wind on the increase of the heat loss through the walls, especially in the corners. Wind washing is a serious problem in the corners of the building because the pressure gradient force of the wind, which drives this phenomenon, is considerable in these areas [28]. Doggett and Brunjes [29] proved that wind washing may significantly increase the heat loss through the walls (up to 42%). Moreover, wind direction [30] and velocity acting upon the building influence the heat loss level. Huifen et al. [31] claimed that the lowest level of heat loss through the external wall was obtained at the wind direction equaling 15°. Shang-Ying and coauthors [32] investigated the influence of wind speed and wind incidence angle (0°, 30°, 60°, 90°) on the heat losses of a thermally insulated, fully open cylindrical cavity with a heated wall. They proved that the average temperature of the heated wall decreases with increasing wind speed and incidence angle. They also showed that the convection heat loss increases along with wind speed, while radiation and conduction heat losses decrease for all wind incidence angles. Wind pressure is also a significant factor which has an effect on the thermal parameters of the wall [33]. Wind pressure against the building results from the air flowing around the building. Wind direction and velocity as well as topography are the factors which have an effect on the wind pressure magnitude [4].

Knowledge of the changes in the thermal resistance of a wall resulting from wind operation (infiltration and wind washing) is crucial for the practical calculation of the heat loss with varying pressure force. The reduction in the value of the thermal resistance can take place only after a certain time of wind operation on the wall and may progress in time. Furthermore, it is important when the thermal parameters of the wall will return to the state of equilibrium after the effect of wind ceases.

The article is focused on the thermal parameters of timber frame wall insulated with mineral wool, wood wool and hemp fibers in a loose form. The purpose of the article was to perform the thermal simulations of different variants of timber frame wall filled with those insulations using the Control Volume Method in the Delphin 5.8 software, which is a common tool for measuring the air moisture and heat performance of building materials and boundaries [34,35,36]. The influence of wind pressure and the time of its impact on the thermal performance of a wall insulated with 150 and 250 mm thick fiber material was considered. The simulations were based on the results of the material properties (thermal conductivity and air permeability as a function of material density) examined in the laboratory that were published in [3,37]. The novelty of the article is a description of the characteristics of fibrous insulation materials of plant origin subjected to various models of air load (air filtration and wind washing) and the consolidation of the results with the characteristics of the traditional insulation material, i.e., mineral wool. The materials of natural origin are increasingly noticeable on the construction market. Investors expect ecological materials which are minimally processed. The research presented in the article may prove helpful in assessing the suitability of the described materials for thermal insulation.

## 2. Materials and Methods

### 2.1. Description of Materials

For the purpose of the study, three different fibrous materials in loose state were chosen: hemp fibers, mineral wool and wood wool. All of them are characterized by a porous structure and thin fibers. Hemp fibers derived from industrial hemp of the “Białobrzeskie” variety were used. The fibers were cut to a length of around 100 mm and were not subjected to additional treatment—combing, cleaning. These fibers were obtained directly in the decortication process of hemp stalks. The fiber thickness was regular, in the range of 40–90 μm. The commercial mineral wool used in the study was characterized by a fiber diameter of 1–11 µm and the commercial wood wool by a diameter of 1–40 μm [38].

**Figure 1:**
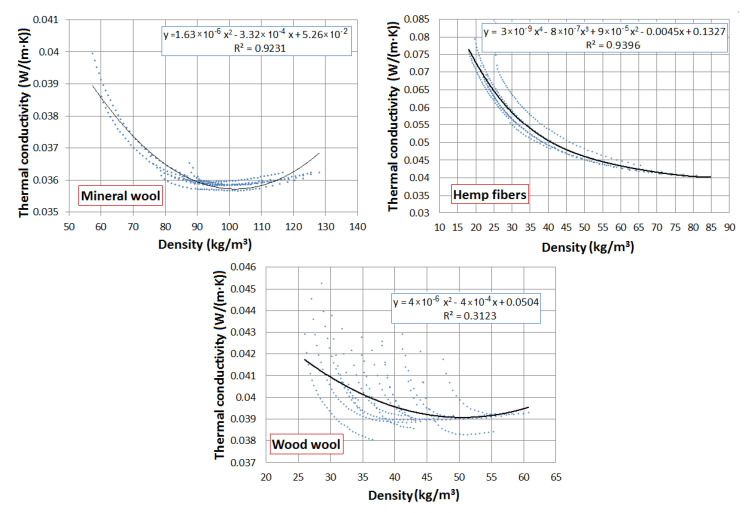
Thermal conductivity of the analyzed materials as a function of density [3,37].

The properties of these materials were investigated in the laboratory and are presented in the works [3,37]. Figure 1 shows the thermal conductivity of the materials as a function of density. It can be observed that in the case of mineral wool and wood wool, the function decreases with the density increase, but after reaching the minimum, it increases. It can be explained that in higher densities, the contact between the fibers plays an important role in the thermal conductivity. Such a situation was not observed in the case of hemp fibers. The higher thickness of the hemp fibers and lack of dust between them did not allow compressing of the material to higher density than 85 kg/m^3^.

**Figure 2:**
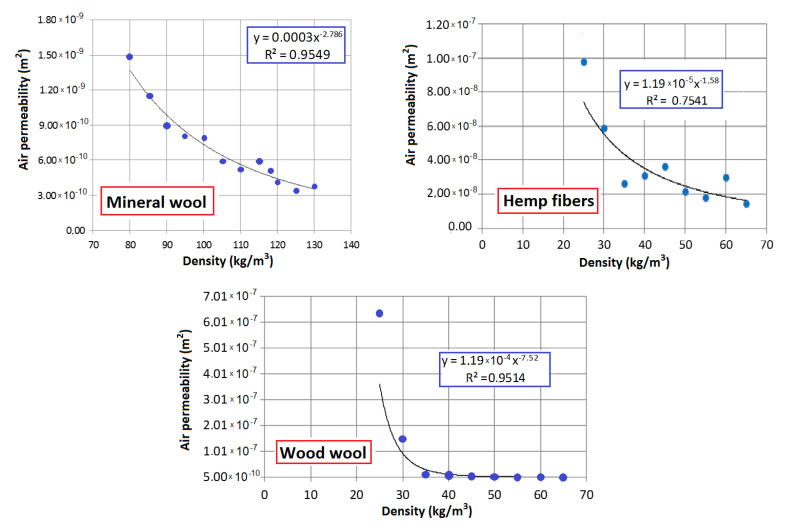
Air permeability of the analyzed materials as a function of density [3,37].

Figure 2 presents the air permeability of these materials as a function of density. It can be seen that the functions decrease with increasing density. In the investigated ranges, the air permeability for mineral wool decreased from 1.48 × 10^−9^ m^2^ for 80 kg/m^3^ to 3.80 × 10^−10^ m^2^ for 130 kg/m^3^, for wood wool from 6.36 × 10^−7^ m^2^ for 25 kg/m^3^ to 5.44 × 10^−10^ m^2^ for 65 kg/m^3^ and for hemp fibers from 9.78 × 10^−8^ m^2^ for 25 kg/m^3^ to 1.05 × 10^−8^ m^2^ for 70 kg/m^3^. The air permeability of the mineral and wood wool is lower due to the occurrence of dust fraction between the fibers.

For the purpose of the simulation, three different densities of each material were chosen (Table 1). The method of selecting the middle densities was based on choosing the ones corresponding to the lowest thermal conductivities. In the case of hemp fibers, this was the middle of the range of the material density. The lowest and highest values of densities chosen for simulation correspond to the natural-sized laboratory models filled with these materials.

### 2.2. Simulation

**Figure 3:**
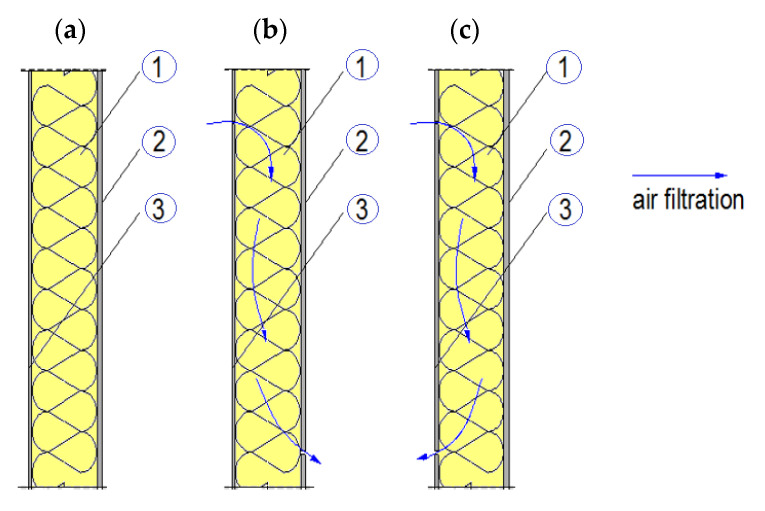
Frame wall model for the simulation: (**a**) no air filtration, (**b**) air filtration, (**c**) wind washing. Where 1—thermal insulation, 2—internal sheathing (gypsum board), 3—external sheathing (OSB).

The simulation was performed using the Control Volume Method implemented in the Delphin 5.8 software (Institute of Building Climatology, Dresden, Germany). The thermal insulation properties were adopted on the basis of the laboratory measurements presented in [3,37]. The simulation model of the frame wall filled with loose thermal insulation is presented in Figure 3. The model consists of OSB as external sheathing (20 mm), thermal insulation—loose mineral wool or hemp fibers or loose wood wool (various: 150, 250 mm)—and gypsum board as internal sheathing (12 mm). The height of the model was 1000 mm. In order to simulate the air movement in the models loaded with the pressure difference of 4 mm, air leaks in the boards were left 96 mm from the top and from the bottom. The models with 150 mm thermal insulation were discretized into 3240 elements and those with 250 mm insulation into 5400 elements.

Three situations were analyzed (Figure 3): only thermal conductivity without air filtration (a), air filtration through the entire model (b), wind washing (c). The models were analyzed under various pressure differences, namely 5, 10 and 15 Pa, which corresponds to wind speed around 2.9, 4.1 and 5.0 m/s [39]. The air pressure values were chosen because of the frequency of wind occurrence in the range of 2–5 m/s, which, in most Polish cities, is over 60% [40].

Table 2 presents the air flows through the simulated 4 mm wide and 1000 mm long leakage. The table presents both the air flows through a wall without insulation and with analyzed insulation materials. For each material, except wood wool, two values are presented: for the higher and lower density. It should be noted that the occurrence of material always decreases the amount of air which filtrates through the element. The lower the air permeability of the insulation, the lower air filtration is possible. It should be noted that wood wool, due to its air flow characteristic (Figure 2), differs from the other materials. The wood wool wall samples with lowest analyzed density can be considered as very leaky and completely non-resistant to air filtration. This is why Table 2 presents in brackets the air flows through wood wool insulation for mid analyzed density.

It could be easily analyzed that, while the air flow through the analyzed leakage in the case of mineral wool will result in around 0.7–2.5 m^3^/h with 50 Pa difference, for hemp fibers, it will reach around 23.3–44.5 m^3^/h, whereas in the case of wood wool in 0.8–2.7 m^3^/h for the densities over 51 kg/m^3^ and up to 106.2 m^3^/h for the density of 30 kg/m^3^. Such air flows can dramatically change the n_50_ factor in the case of natural fibrous insulation, while with mineral wool, it will change only to a limited extent. However, it should be noted that n_50_ is an important factor when investigating air infiltration but gives no information about wind washing [41].

The boundary conditions were set as internal temperature 20 °C, external temperature −20 °C; the exchange coefficients for heat flow were set as default in ISO 6949: 8 W/(m^2^·K) from inside and 25 W/(m^2^·K) from outside. The authors did not differ the exchange coefficient from outside regarding the air speed. The pressure loads were activated after thermal equilibrium of the model, for various times, from 30 to 180 min, with time step of 30 min. The authors opted for short periods of pressure load, because only long periods have been analyzed so far [41]. The aim of the simulation was to establish the reaction time of the model on the pressure load and time of the model to return to equilibrium state. Simultaneously, the reduction of thermal resistance due to air filtration was calculated. The R-value reduction was calculated as a ratio between the no air filtration case as a reference and the case of air filtration case or wind washing, respectively.

## 3. Results and Discussion

**Figure 4:**
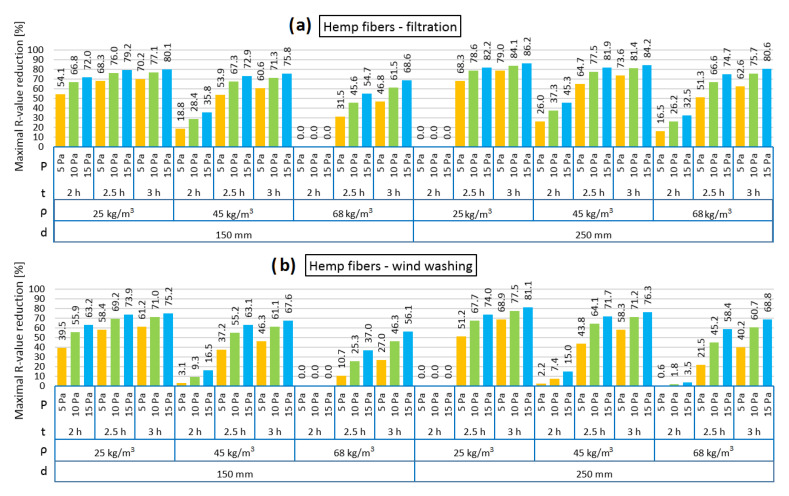
Maximal R-value reduction for hemp fibers as loose-fill thermal insulation: air filtration (**a**), wind washing (**b**).

**Figure 5:**
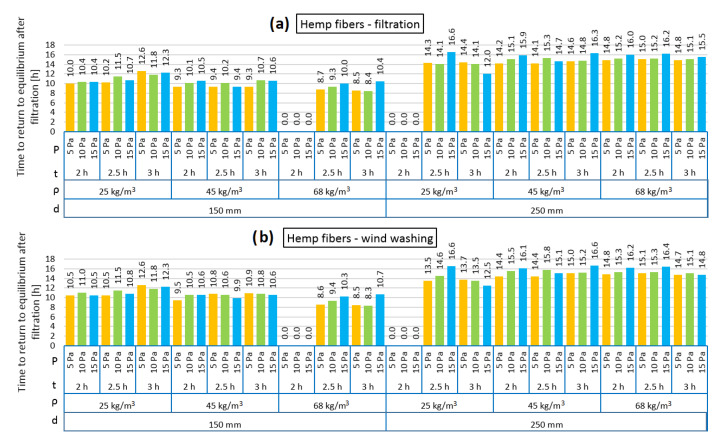
Time to return to equilibrium after filtration for hemp fibers as loose-fill thermal insulation: air filtration (**a**), wind washing (**b**).

**Figure 6:**
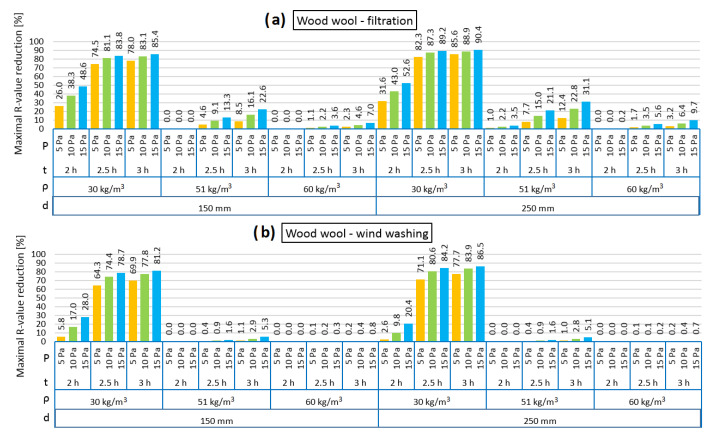
Maximal R-value reduction for wood wool as loose-fill thermal insulation: air filtration (**a**), wind washing (**b**).

**Figure 7:**
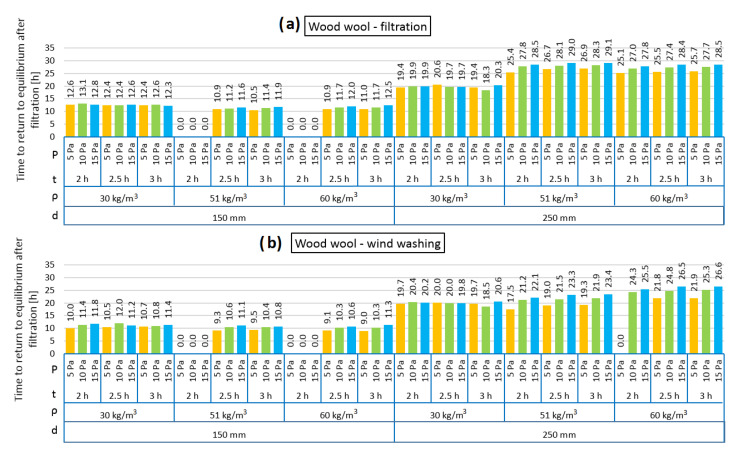
Time to return to equilibrium after filtration for wood wool as loose-fill thermal insulation: air filtration (**a**), wind washing (**b**).

**Figure 8:**
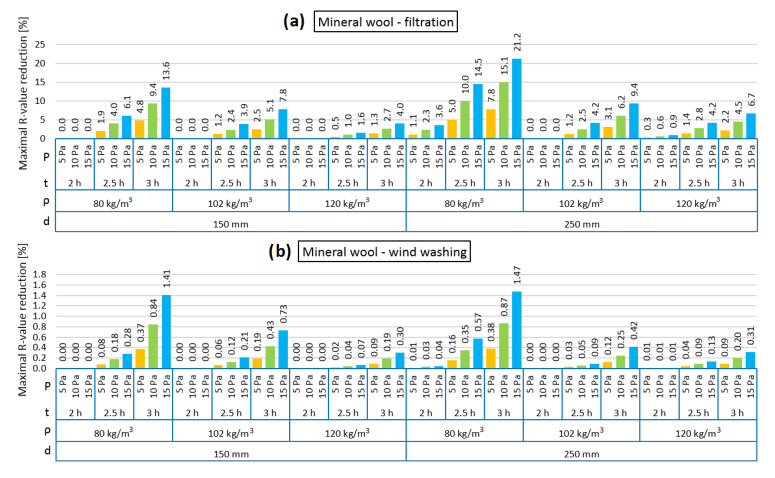
Maximal R-value reduction for mineral wool as loose-fill thermal insulation: air filtration (**a**), wind washing (**b**).

**Figure 9:**
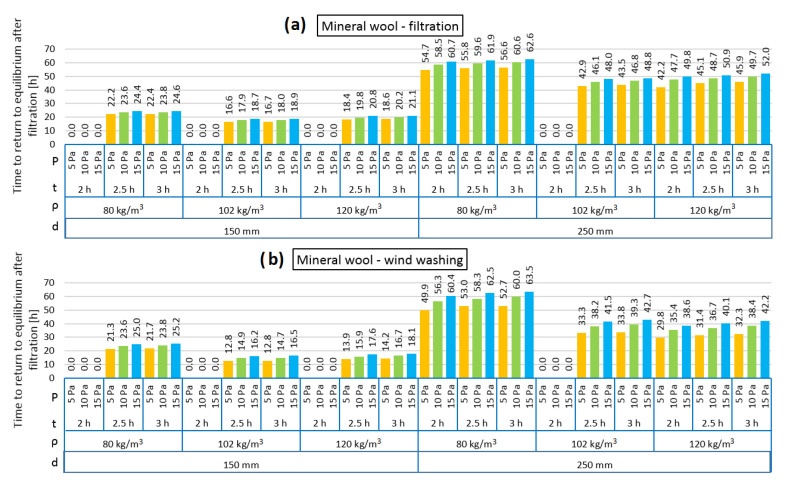
Time to return to equilibrium after filtration for mineral wool as loose-fill thermal insulation: air filtration (**a**), wind washing (**b**).

Figure 4, Figure 5, Figure 6, Figure 7, Figure 8 and Figure 9 show the results obtained in the simulation: maximal R-value reduction and time to return to equilibrium after filtration for walls insulated with three different insulations. Figures contain symbols referring to: “P”—wind pressure (Pa) “t”—time of wind operation (h), “ρ”—density of the material (kg/m^3^), “d”—thickness of the insulation layer (mm).

Figure 4 presents the thermal resistance (R) reduction of the models filled with loose hemp fibers in the cases of air infiltration and wind washing. It should be noted that the reduction was noticeable only after 102–128 min of pressure load. At least a few dependencies can be noticed: the heat losses caused by the air flow increase along with the pressure difference, and the heat losses increase with the extension of the pressure effect. Moreover, with increasing density of insulation, the air permeance decreases and, thus, the heat losses via air filtration are smaller. Additionally, the heat losses in the wind washing model are slightly smaller than in the air filtration model. It can be observed that both for the air filtration and wind washing models, the heat losses are slightly greater in 250 mm than in 150 mm, respectively. A special case, however, is the 250 mm model with density of 25 kg/m^3^, where no heat losses due to air filtration or wind washing with a 2-h exposure to pressure load were found. This may be due to the simulation process itself and the solver calculations. Additionally, 2 h of pressure load also did not change R in the case of the 150 mm model filled with hemp fibers of 68 kg/m^3^ density, which can be explained by partial resistance to wind filtration in such a short time in the case of the higher density of the insulation material.

Thus, the lowest R reduction in the 150 mm thickness of insulation in the air infiltration model is observed for 68 kg/m^3^, 2 h of 5 Pa load, while the highest is observed for 25 kg/m^3^, 3 h of 15 Pa load. The lowest and highest R reduction in the 250 mm thickness of insulation in the air infiltration model is observed for 25 kg/m^3^ density, the lowest for 2 h of 5 Pa load and the highest for 3 h of 15 Pa load.

The same was observed for the wind washing model.

It is worth noticing that with the increase in the hemp fibers’ density, a longer period of air filtration is needed to cause a similar R reduction. On the example of the 150 mm model with 5 Pa pressure difference, a similar R reduction is caused by 2 h of air filtration in the 25 kg/m^3^ case, 2.5 h in the 45 kg/m^3^ case and more than 3 h in the 68 kg/m^3^ case.

Figure 5 presents the time taken for the models loaded with pressure difference to return to thermal equilibrium. It can be observed that the times for both the air infiltration and wind washing models are comparable. In the 150 mm model, the time varied between 8.4 and 12.3 h and in the 250 mm model between 12.0 h and 16.6 h. Both in the air infiltration and wind washing models with the insulation thickness of 150 mm, the time needed to return to equilibrium is slightly shorter in the case of the higher analyzed hemp fiber density (68 kg/m^3^). Moreover, in the 150 mm model with density of 25 kg/m^3^, the effect of the length of pressure load for the analyzed time can be seen; the longer the load, the longer time needed to return to equilibrium. Such dependences were not observed in the thicker model or for other densities.

Figure 6 presents the R reduction of the models filled with loose wood wool in the cases of air infiltration and wind washing. It should be noted that the reduction was noticeable only after 114–126 min of pressure load. It can be seen that the highest R reduction is observed for the lowest density of insulation (30 kg/m^3^), which is related to the highest air permeability amongst the examined wood wool cases, as shown in Figure 1. The influence of the increase in pressure difference and length of the time of pressure load on the R reduction are clearly seen, both causing higher R reduction.

In the 150 mm 2 h air filtration model, both for 51 kg/m^3^ and 60 kg/m^3^ wood wool density, the R reduction is zero, regardless of the pressure difference, while in the 250 mm model, the R reduction is minimal. In the wind washing case, regardless of the insulation thickness, 2 h of pressure load causes no R reduction for 51 kg/m^3^ and 60 kg/m^3^ wood wool density. Similar as for hemp fibers, wind washing causes a lower R reduction than air filtration, which is especially clearly seen for 2 h pressure load. It was observed that 2 h of air load in the air filtration model causes a 4.5 times greater R reduction in the 150 mm model, while a twelvefold reduction is seen in the 250 mm model, in comparison to the corresponding thickness in the wind washing model.

In the case of wood wool, the material density has a significant influence on the external wall behavior under the influence of wind. This is different in the case of hemp fibers, where for all densities, the reduction in the R value was significant.

Figure 7 presents the time taken for the models loaded with pressure difference to return to thermal equilibrium. It can be observed that the times for both the air infiltration and wind washing models are comparable. In the 150 mm model, the time varied between 9.0 and 13.1 h, while in the 250 mm model, it varied between 17.5 and 29.1 h. There is a noticeably longer time to return to equilibrium for the model with 250 mm insulation. Both in the air infiltration and wind washing models with the insulation thickness of 150 mm, it can be observed that the time needed to return to equilibrium is slightly longer for the lowest density of wood wool of 30 kg/m^3^. Conversely, in the case of 250 mm insulation thickness, the shortest returning time is observed for the lowest density. Different lengths of wind operation result in almost the same values of time to return to equilibrium after filtration for both the shortest and the longest presented operation time. The dependency is perceptible on the pressure load. In all analyzed cases, the influence of the pressure load and difference in the returning time is clearly seen. The higher the pressure load, the longer the time to return to thermal equilibrium.

Figure 8 presents the R reduction of the models filled with loose mineral wool in the cases of air infiltration and wind washing. It should be noted that the reduction was noticeable only after 110–132 min of pressure load. Both in the air infiltration and wind washing models, the highest R reduction is observed for the case of the lowest mineral wool density (80 kg/m^3^) and this is related to the highest air permeability for mineral wool with a density of 80 kg/m^3^, as shown in Figure 1. It can be observed that, in each case, the R reduction was higher in the 250 mm model than for 150 mm. Both in the air filtration and wind washing models, 2 h of air load does not change the thermal resistance of the wall filled with 102 kg/m^3^ loose mineral wool. It is the same for 150 mm model insulated with mineral wool with a density of 120 kg/m^3^, but in the 250 mm model, the R reduction is minimal. While the relation between density and air permeability of mineral wool has a lowering function, the relation between density and thermal conductivity is second-degree polynomial with a minimum of around 102 kg/m^3^. This may be the reason that 2 h of air load cause changes in the model of higher density.

Figure 9 presents the time taken for the models loaded with pressure difference to return to thermal equilibrium. Although the R reduction was not high, the time taken to return to equilibrium was long. It can be observed that in the cases of both air infiltration and wind washing, a longer returning time was clearly needed in the 250 mm model. In the 150 mm model, the time varied between 12.8 and 25.2 h and in the 250 mm model between 29.8 and 63.5 h. The longest time was needed in the case of the thermal insulation made of the mineral wool with the lowest density (80 kg/m^3^). In all analyzed cases, the influence of the pressure load difference on the returning time is clearly seen. The higher the pressure load, the longer the time needed to return to thermal equilibrium.

In the case of mineral wool, air filtration has a weaker effect on reducing the thermal resistance of the external wall in comparison to insulation based on natural materials. The impact of wind washing is much lower than filtration. In other studies, the influence of wind washing [28] on the reduction of the R value was examined. Wind speed was used as a variable. With a wind speed of 1 m/s and mineral wool insulation with a thickness of 50 mm and with a density of 70 kg/m^3^, the R value was reduced by 0.03 m^2^K/W. In other studies [42], the IR analysis of the wall insulated with loose mineral wool of low density, based on surface and surrounding air temperature comparison, revealed a 75% reduction in the thermal resistance of the measured element, caused by wind washing, which can be translated into a four-fold increase in the heat transfer coefficient. This proves that wind washing may lead to high heat losses in elements filled with a low density of loose mineral wool.

## 4. Conclusions

This paper presents an analysis pertaining to the thermal parameters exhibited by a timber frame wall which was insulated by means of mineral wool, wood wool and loose hemp fibers. The effects of wind pressure and the time of its influence on a wall insulated by means of fiber material with a thickness of 150 mm as well as 250 mm were investigated in the conducted research.

A thorough analysis of the obtained results enables us to formulate the following conclusions:

- The reduction in thermal resistance (R) in the analyzed cases depends on the density of thermal insulation, its thickness, but also time and pressure load. With increasing density, the R reduction is lower due to the lower air permeability of the material (both air filtration and wind washing cases). The most remarkable influence of insulation material density on the R value reduction can be observed in the case of wood wool. Wind washing causes lower R reduction than air filtration in all models.
- The longer the time and the higher the pressure load, the higher the R reduction. Higher R reduction was observed in the thicker models. After 2 h of exposure of the mineral wool model to air pressure, the reduction in the R value is clearly smaller (equal to zero in almost all cases) in comparison to 2.5 and 3 h of exposure. In the walls insulated with natural fibers, these differences are less visible (air filtration case), especially in the case of hemp fibers (thickness 150 mm, density 25 kg/m^3^).
- The period needed for the models after air filtration (wind washing as well) to return to the equilibrium state depends strongly on the thermal insulation thickness. The higher the thickness, the longer the time. The period is comparable for both air filtration and wind washing. In the case of mineral wool, the time to return to equilibrium after filtration increases along with wind pressure and the time period of its exposure. There are no such clear relationships in the case of the walls insulated with natural fibers.
- It can be clearly seen that the R reduction depends on the time of the pressure load and that a 3 h period does not enable the maximum change. The intention was to identify the results of short periods, which occur quite often in the Polish climate due to wind operation. It should be also mentioned that the authors did not analyze thermal buoyancy, which will increase the convection in the thermal insulation layer. The time period of returning to the equilibrium state is three or four times longer than the time period of pressure load in the case of the 150 mm model but up to ten times longer in the 250 mm model (wood wool and hemp fibers) or twenty times longer (mineral wool).
- The difference between the natural and the synthetic fiber materials can be identified mostly in the air transport properties. Mineral wool is characterized by low air permeability even for the lower analyzed densities, in contract to natural materials. Air permeability as a function of wood wool density, due to the dusty fraction content, is characterized by a rapidly decreasing shape. Hemp fibers, because of the preparation process, are devoid of the dusty fraction and this is why this material is the most porous amongst those analyzed. These properties were presented in the results of the simulation; hemp fibers were characterized by the strongest susceptibility to air filtration, and wood wool was also high, but lower than hemp fibers, while mineral wool displayed the lowest values.
- It should be noted that the numerical software calculations are based on the simplification of the material properties. Unlike in reality, the air permeability and thermal conductivity of fiber materials were the same in every area of the model. Almost always, there are gaps between the fibers and the filled frame; thus, the risk of air filtration in the insulation material is higher. As a result, the heat loses in such elements are higher than in the simulations.

Currently, the authors are focused on the hygrothermal analysis of natural fiber materials for thermal insulation. New plant materials are being tested for the base transport parameters. A laboratory investigation focused on the hygrothermal state in natural-sized models filled with fiber materials is being conducted simultaneously.

## Figures and Tables

**Table 1:** Parameters of analyzed materials.

Density (kg/m^3^)	Thermal Conductivity λ (W/(mK))	Air Permeability k (m^2^)
mineral wool		
80	3.64 × 10^−2^	14.84 × 10^−10^
102	3.57 × 10^−2^	6.84 × 10^−10^
120	3.62 × 10^−2^	4.17 × 10^−10^
wood wool		
30	4.09 × 10^−2^	1480.07 × 10^−10^
51	3.90 × 10^−2^	16.91 × 10^−10^
60	3.94 × 10^−2^	4.98 × 10^−10^
hemp fibers		
25	6.49 × 10^−2^	781.31 × 10^−10^
45	4.79 × 10^−2^	286.60 × 10^−10^
68	4.12 × 10^−2^	141.16 × 10^−10^
Gypsum board	13.00 × 10^−2^	-
OSB (oriented strand board)	20.00 × 10^−2^	-

Note: - for non permeable materials

**Table 2:** Air flows through different insulation materials.

Pressure Difference (Pa)	Air Flow (m^3^/h)			
	Without Material	Hemp Fibers	Wood Wool	Mineral Wool
5	41.13	2.33–4.45	0.08–(0.27)–10.62	0.07–0.24
10	58.16	4.66–8.91	0.16–(0.54)–21.23	0.13–0.48
15	71.23	6.70–13.37	0.24–(0.81)–31.85	0.20–0.71
